# Application of Flow Cytometry in the Diagnostics Pipeline of Primary Immunodeficiencies Underlying Disseminated *Talaromyces marneffei* Infection in HIV-Negative Children

**DOI:** 10.3389/fimmu.2019.02189

**Published:** 2019-09-13

**Authors:** Pamela P. Lee, Mongkol Lao-araya, Jing Yang, Koon-Wing Chan, Haiyan Ma, Lim-Cho Pei, Lin Kui, Huawei Mao, Wanling Yang, Xiaodong Zhao, Muthita Trakultivakorn, Yu-Lung Lau

**Affiliations:** ^1^Department of Paediatrics and Adolescent Medicine, Li Ka Shing Faculty of Medicine, The University of Hong Kong, Hong Kong, China; ^2^Department of Pediatrics, The University of Hong Kong-Shenzhen Hospital, Shenzhen, China; ^3^Division of Allergy and Clinical Immunology, Department of Pediatrics, Chiang Mai University, Chiang Mai, Thailand; ^4^Chongqing Key Laboratory of Child Infection and Immunity, Children's Hospital of Chongqing Medical University, Chongqing, China

**Keywords:** *Taloromyces marneffei*, flow cytometry, X-linked hyper-IgM syndrome, CD40L, STAT1, interferon gamma receptor deficiency

## Abstract

*Talaromyces (Penicillium) marneffei* is an AIDS-defining infection in Southeast Asia and is associated with high mortality. It is rare in non-immunosuppressed individuals, especially children. Little is known about host immune response and genetic susceptibility to this endemic fungus. Genetic defects in the interferon-gamma (IFN-γ)/STAT1 signaling pathway, CD40/CD40 ligand- and IL12/IL12-receptor-mediated crosstalk between phagocytes and T-cells, and STAT3-mediated Th17 differentiation have been reported in HIV-negative children with talaromycosis and other endemic mycoses such as histoplasmosis, coccidioidomycosis, and paracoccidioidomycosis. There is a need to design a diagnostic algorithm to evaluate such patients. In this article, we review a cohort of pediatric patients with disseminated talaromycosis referred to the Asian Primary Immunodeficiency Network for genetic diagnosis of PID. Using these illustrative cases, we propose a diagnostics pipeline that begins with immunoglobulin pattern (IgG, IgA, IgM, and IgE) and enumeration of lymphocyte subpopulations (T-, B-, and NK-cells). The former could provide clues for hyper-IgM syndrome and hyper-IgE syndrome. Flow cytometric evaluation of CD40L expression should be performed for patients suspected to have X-linked hyper-IgM syndrome. Defects in interferon-mediated JAK-STAT signaling are evaluated by STAT1 phosphorylation studies by flow cytometry. STAT1 hyperphosphorylation in response to IFN-α or IFN-γ and delayed dephosphorylation is diagnostic for gain-of-function STAT1 disorder, while absent STAT1 phosphorylation in response to IFN-γ but normal response to IFN-α is suggestive of IFN-γ receptor deficiency. This simple and rapid diagnostic algorithm will be useful in guiding genetic studies for patients with disseminated talaromycosis requiring immunological investigations.

## Introduction

*Talaromyces marneffei* (previously known as *Penicillium marneffei*) is a pathogenic fungus indigenous to Southeast Asia (1–5). Before the human immunodeficiency virus (HIV) epidemic, *T. marneffei* was an extremely rare pathogen in humans (1). Since the late 1980s, talaromycosis emerged as a clinically important opportunistic infection following the exponential growth in the incidence of HIV in Southeast Asia, especially in Northern Thailand, Vietnam, Guangxi, and Guangdong in Southern China (2–6). An increasing number of cases have also been reported in Myanmar, Laos, Cambodia, Singapore, Malaysia, Indonesia, and northeastern India (7, 8). *T. marneffei* infection is classified as an acquired immunodeficiency syndrome (AIDS)-defining illness and listed as one of the HIV clinical stage 4 conditions (6). The trend of *T. marneffei* infection closely paralleled that of HIV, and in areas where reduction of HIV transmission and availability of highly active antiretroviral therapy (HAART) have improved, a decrease in the prevalence of *T. marneffei* infection has been observed (9, 10). A similar trend is also observed in endemic mycoses caused by other thermally dimorphic fungi such as coccidioidomycosis and histoplasmosis (11, 12). The close relationship between disease manifestation and severity with CD4+ cell count confirms the central importance of cell-mediated immunity against endemic fungi.

While the vast majority of talaromycosis were reported in patients with AIDS, a smaller proportion of cases were described in patients with hematological malignancies, autoimmune diseases, and diabetes mellitus and renal or hematopoietic stem cell transplant recipients (13, 14). Autoantibody against IFN-γ has been reported to be associated with adult-onset immunodeficiency in patients of Asian ethnicity, resulting in predisposition to talaromycosis, melioidosis, salmonellosis, and non-tuberculous mycobacterial infections (15–20). Talaromycosis in otherwise healthy children is uncommon. We performed a systematic literature review of 509 reports on human *T. marneffei* infection published between 1950 and 2011, and identified 32 patients aged 3 months to 16 years with no known HIV infection. Twenty-four patients (75%) had disseminated disease, and 55% died of talaromycosis. Eight patients, all reported prior to 2010, had some forms of immunodeficiencies which were not genetically defined (hypogammaglobulinemia, CD4 lymphopenia, common variable immunodeficiency, Kostmann syndrome, and clinically probable X-linked hyper-IgM syndrome) or blood disorders such as aplastic anemia. Four others had abnormal immune functions while immune evaluation was not performed for the rest (21). In 2014, we discovered gain-of-function (GOF) STAT1 disorder as the underlying cause of disseminated talaromycosis in 3 pediatric patients in Hong Kong (22). Recently, primary immunodeficiencies (PID) in HIV-negative children with *T. marneffei* infection have been increasingly recognized, including CD40L deficiency and autosomal dominant (AD) hyper-IgE syndrome (23–29). However, talaromycosis as an indicator of underlying PID in HIV-negative children is still under-recognized, as diagnostic immunological evaluations remained limited in many recently published cases (30–34).

The close epidemiological relationship between HIV and *T. marneffei*, and the fact that talaromycosis is an AIDS-defining illness (6) suggests that individuals who are HIV negative and without secondary immunodeficiencies may have underlying immune defects that are unrecognized. There is a need to adopt a systematic approach to evaluate HIV-negative individuals with talaromycosis, by performing stepwise immunological investigations to guide confirmatory genetic tests, targeting on disorders affecting IFN-γ mediated crosstalk between phagocytic cells and T-lymphocytes, and signaling pathways involved in T-helper 1 (Th1) and Th17 differentiation. In this article, we illustrate how flow cytometric evaluation can be incorporated into a simple and rapid diagnostic algorithm for patients with disseminated talaromycosis requiring immunological investigations.

## Methodology

### Patients

The Asian Primary Immunodeficiency (APID) Network was established by the Department of Pediatrics and Adolescent Medicine, The University of Hong Kong (35). Since 2001, 1,599 patients with suspected PID from more than 90 centers in 13 countries and regions in Asia were referred to the APID Network for genetic studies. Among them, eight patients had *T. marneffei* infection including four from Hong Kong, two from Southern China and two from Northern Thailand. Clinical features and immunological parameters were retrieved from the database. Consent for genetic diagnosis and functional study was obtained from parents, and the study was approved by the Institutional Review Board of The University of Hong Kong/Hospital Authority Hong Kong West Cluster.

### Flow Cytometric Evaluation of CD40 Ligand (CD40L) Expression

Detailed methodology was previously reported by An et al. (36) and Du et al. (29). Briefly, peripheral blood mononuclear cells (PBMC) obtained from patients and healthy controls were isolated by ficoll-hypaque density gradient centrifugation. At least 1 × 10^6^ PBMCs were cultured at 37°C for 4 h at 500 μl RPMI 1640 medium supplemented with 10% heat-inactivated fetal calf serum (FCS), and activated by 50 ng/ml phorbol myristate acetate (PMA) and 500 ng/ml ionomycin (Sigma, Shanghai, China). Cells were collected, washed and incubated with PerCP-Cy5.5-conjugated anti-human CD3 (mouse IgG1, κ, clone OKT3), FITC-conjugated anti-human CD8 (mouse IgG1, κ, clone RPA-T8), and PE-conjugated anti-human CD154 antibody (mouse IgG1, κ, clone 24-31) or PE-conjugated isotype control (IgG1, clone P3). All antibodies were obtained from eBioscience (San Diego, CA, USA). Flow cytometric analysis was performed (FACSCanto II, BD Biosciences), gating on live cells determined by scatter characteristics. Data was analyzed using FlowJo software (Tree Star, Ashland, OR, USA). The percentage of CD3+CD8-CD154+ cells was determined by gating on dot-plot histograms and comparing with cells stained with isotype control reagents.

### Flow Cytometric Quantification of STAT1 Phosphorylation

Detailed methodology was previously published by our group (22). 10^6^ PBMCs were stimulated with recombinant human IFN-α (40,000 IU/ml) or IFN-γ (5,000 IU/ml) for 20 or 30 min as indicated. To study the kinetics of STAT1 dephosphorylation, cells were further treated with staurosporine (500 nM) for 30 min. Cells were washed and stained with FITC-conjugated anti-human CD3 and pacific-blue-conjugated anti-human CD14. This was followed by fixation with BD Phosflow™ Fix Buffer I and permeabilization in BD Phosflow™ Perm Buffer III. After wash, cells were stained with AlexaFluor® 647-conjugated anti-human STAT1 (pY701) for intracellular phosphorylated STAT1 (pSTAT1). The percentage of intracellular pSTAT1 expression and mean fluorescent intensity (MFI) in CD3+ T-cells and CD14+ monocytes were determined by using flow cytometry and analyzed by FlowJo (version: 8.8.2). Gating strategy is shown in Supplementary Figure 1.

### Sanger Sequencing

Genomic DNA was isolated from peripheral blood obtained from the subjects. Fiffty nanograms DNA were added to sequencing primers for human *STAT1, CD40LG*, and *IFNGR1* genes for sequence analysis (see Supplementary Table 1 for primer sequences) using Applied Biosystems 3730xl DNA Analyzer. Sequence analysis with reference sequence of the corresponding genes was performed using the National Center for Biotechnology Information program Basic Local Alignment Search Tool (https://blast.ncbi.nlm.nih.gov/Blast.cgi/).

### Whole Exome Sequencing Procedure

Three microgram of genomic DNA extracted from the patient's PBMCs for exon capture by using Agilent SureSelect Human All Exon 50 Mb and library preparation, according to standard procedures. WES was performed using Illumina HiSeq 2000 (Illumina, Inc., San Diego, CA 92122 USA) on genomic DNA enriched for exonic fragments using Agilent SureSelect V3 (Agilent technologies, Santa Clara, CA 95051 USA). The data were processed using GATK. Briefly, paired-end reads were mapped to human reference genome (GRCh37/hg19) using Burrows-Wheeler Aligner (http://bio-bwa.sourceforge.net/). Picard was used to mark duplicated reads and Realigner Target Creator and Base Recalibrator of GATK were used for realignment and base quality recalibration. Single nucleotide variants (SNVs) were called by using GATK (http://www.broadinstitute.org/gatk/), and indel were identified by using GATKindel, Dindel (http://www.sanger.ac.uk/resources/software/dindel/), and Pindel (https://trac.nbic.nl/pindel/). SNVs with minor allele frequency of 1% are considered as polymorphism. SNV calls with a quality phred score of more than 30, mapping quality phred score of more than 10, and coverage depth of more than 10 were kept for further analysis. Mutations were annotated using annovar. Of the SNV calls, their population frequency in dbSNP, 1000 Genome Project, NHLBI Exome Sequencing Project (ESP) ESP6500 data set (http://evs.gs.washington.edu/EVS/), and an internal database on exome sequencing was examined and relatively common variants considered unlikely to be related to the disease phenotype were excluded from further consideration. Potential functional impact of the missense mutations was also evaluated by algorithms including SIFT, PolyPhen2, LJB_PolyP, LJB_MutationTaster, LJB_LRT, and those considered to be unlikely to have a strong functional impact on the protein structure and function were also removed from further consideration. Variants that were considered high (frame shift mutations, splicing site mutations, start/stop gains and losses) and moderate functional impact (missense substitutions and non-frame shift insertion deletions) were further considered. The putative disease-causing variants were confirmed by Sanger sequencing.

## Results

### Family 1

**Figure 1:**
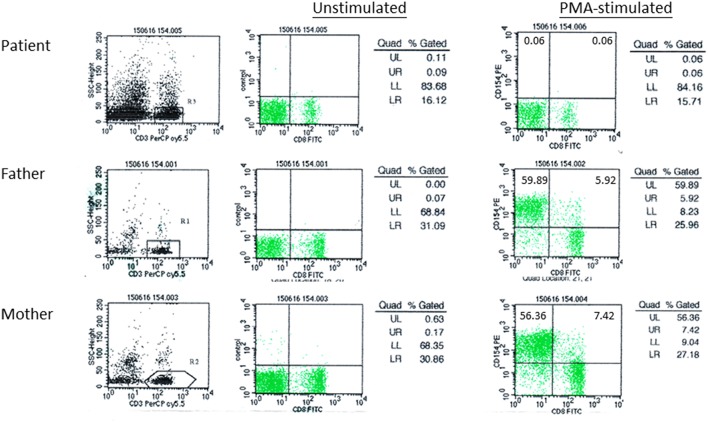
CD40L expression in patient F1 and his parents. Peripheral blood mononuclear cells (PBMCs) were activated by phorbol myristate acetate (PMA) and ionomycin, followed by immunostaining with anti-CD3-PerCP-Cy5.5, anti-CD8-FITC, and anti-CD154-PE antibody. Flow cytometric analysis was performed gating on CD3+ T-cells. CD3+CD8-CD154+, represented by UL (upper left quadrant), indicated activated T-cells expressing CD40L.

**Figure 2:**
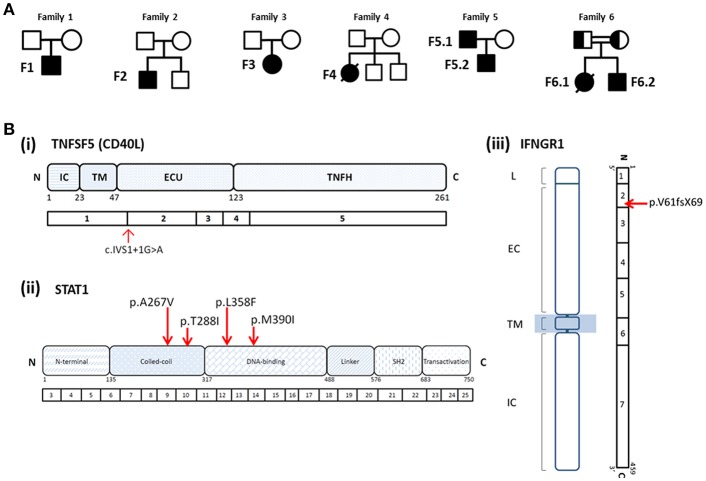
**(A)** Pedigrees of Families 1–6. **(B)** Mutations identified in patients with *T. marneffei* infections. (i) Splice site mutation in *TNFSF5* (*CD40L*) in Patient F1; (ii) heterozygous missense mutations in *STAT1* identified in Patients F2 (p.A267V), F3 (p.T288I), F4 (p.L358F), F5.1, and F5.2 (p.M390I); (iii) homozygous mutation in *IFNGR1* identified in Patient F6.2 (p.V61fsX69) and his deceased elder sister (F6.1). Both parents were found to be heterozygous carriers of the mutation.

A 29-month old boy (F1) was admitted because of persistent fever for 1 month and neck mass for 10 days. Chest X-ray showed pneumonic changes. Cervical lymph node biopsy and endobronchial biopsy both yielded *T. marneffei*. Blood culture was positive for *T. marneffei* and *Staphylococcus epidermidis*. He has a past history of recurrent infections since 13 months of age. He received BCG vaccination at birth, and developed left axillary lymphadenitis which resolved spontaneously. His complete blood count was unremarkable. Lymphocyte subset showed reduced T and NK cells, and Immunoglobulin profile showed reduced serum IgG (431 mg/dl), lowish IgA (44 mg/dl), and IgM (62mg/dl; Table 1). HIV serology was negative. CD40L expression on CD3+ T-cells activated by phorbol myristate acetate (PMA) and ionomycin was absent in the patient, and normal in both parents (Figure 1). X-linked hyper-IgM syndrome was genetically confirmed by the identification of a mutation in the *TNFSF5* (*CD40L*) gene, g.IVS1+1G>A predictive of aberrant splicing (Figure 2). The episode of disseminated talaromycosis was treated with voriconazole for 4 months. He was put on trimethoprim sulfamethoxazole prophylaxis and continued with monthly immunoglobulin replacement. Two years later, he developed persistent sinusitis and culture from nasal secretions again yielded *T. marneffei*. He was treated with a short course of voriconazole.

**Table 1:** Hematological and immunological parameters.

	**F1**	**F2**	**F3**	**F4**	**F5.1**	**F6.1**
Hb (g/dl)	12.0	10.5	10.3	9.8	8.8	6.9
**Full Blood Count**						
WCC (× 10^9^/l)	2.37	6.3	12.81	6.5	3.2	24.5
ANC (× 10^9^/l)	0.87	4.86	10.71	2.86	N/A	18.6
ALC (× 10^9^/l)	1.28	0.84	0.89	2.87	N/A	4.94
PLT (× 10^9^/l)	367	308	229	297	N/A	24
ESR (mm/h)	44	75	104	89	N/A	N/A
CRP (mg/l)	3	24.5	6.75	5.67	N/A	N/A
**Serum Ig**						
IgG (g/l)	4.3 (6.76–13.49)	26.8 (5.37–16.82)	32.1 (7.24–13.8)	11.13 (7.24–13.8)	N/A	18.8
IgA (g/l)	0.44 (0.63–2.34)	4.09 (0.74–2.61)	3.30 (0.68–2.29)	0.74 (0.68–2.29)	N/A	2.43
IgM (g/l)	0.62 (0.64–2.37)	1.23 (0.40–1.95)	0.97 (0.88–2.75)	1.59 (0.88–2.75)	N/A	2.61
**Lymphocyte Subset**						
CD3+ (/μl, %)	706 (55.2)	841 (78.1)	833 (76.2)	1832 (63.7)	N/A	3276 (66.2%)
Normal range for age and sex	1,500–2,900 (62–70)	1,100–2,200 (56–72)	1,300–2,200 (64–72.5)	1,300–2,200 (64–72.5)		(50–81)
CD4+ (/μl, %)	454 (35.5)	297 (27.6)	375 (34.3)	897 (31.2)	93 (29%)	2,153 (43.5)
Normal range for age and sex	1,000–2,100 (29–40)	600–1,600 (27–34)	600–1,100 (29.5–35.5)	600–1,100 (29.5–35.5)	600–1,100 (29.5–35.5)	(22-50)
CD8+ (/μl, %)	228 (17.8)	400 (37.1)	392 (35.8)	701 (24.4)	138 (43%)	1,064 (21.5%)
Normal range for age and sex	700–1,100 (19–25)	500–1,200 (23–30)	500–1,000 (24–33.5)	500–1,000 (24–33.5)	500–1,200 (23–30)	(18–44%)
CD19+ (/μl, %)	512 (40.0)	158 (14.7)	189 (17.2)	879 (30.6)	Normal	1168 (23.6%)
Normal range for age and sex	500–1,200 (18.5–28)	200–600 (15–20)	300–500 (14–21)	300–500 (14–21)	200–600 (15–20)	(7–27%)
CD16/56+ (/μl, %)	40 (3.1)	52 (4.9)	25 (2.3)	101 (3.5)	N/A	190 (9.9%)
Normal range for age and sex	300–600 (9–195)	300–600 (11–24)	300–500 (11–23)	300–500 (11–23)		(2–40%)

At 6 years, he developed fever, cough, stridor and shortness of breath. Laryngoscopy showed vocal cord swelling, and he was subsequently intubated and ventilated for acute upper airway obstruction. Throat swab and sputum yielded positive growth of *T. marneffei*. CT scan showed thickened vocal cord and laryngeal narrowing. There was segmental collapse and consolidation in the left lung, and calcified lymph nodes in the bilateral hilar and mediastinal regions. There was clinical improvement after adding on anti-fungal treatment and he was extubated 3 days later. He completed a 6-week course of voriconazole and had good clinical recovery, and was put on fluconazole prophylaxis. A 10/10 matched-unrelated donor was identified and he is currently prepared for haematopoietic stem cell transplantation.

### Family 2, 3, and 4

We previously reported 3 pediatric patients with *T. marneffei* infection and chronic mucocutaneous candidiasis (CMC) caused by GOF *STAT1* mutations (22). Briefly, F2 presented with cervical and mediastinal lymphadenopathy at 15 years and fine needle aspiration of the cervical lymph node yielded *T. marneffei*. F3 presented with pneumonia and otitis externa at the age of 7 years. Nasopharyngeal aspirate was positive for influenza A with prolonged carriage. Computed tomography (CT) of the thorax showed mediastinal and hilar lymphadenopathy with multiple thin-walled pulmonary cystic cavities. Bronchoalveolar lavage yielded *T. marneffei* and cytomegalovirus. Ear swab culture was positive for *Candida albicans* and *C. tropicalis*. Both F2 and F3 received amphotericin treatment with good response, and were put on long-term itraconazole prophylaxis without disease recurrence.

F4 presented with fever and cervical lymphadenopathy at 5 years old. Lymph node biopsy yielded *Mycobacterium tuberculosis* and *T. marneffei*. She received anti-tuberculosis treatment and itraconazole with good response. At 16 years old, she developed invasive aspergillosis and haemophagocytic syndrome, and died of massive pulmonary hemorrhage.

Immunological parameters were summarized in Table 1. Whole exome sequencing revealed the presence of heterozygous mutation in *STAT1* gene in the coiled-coil domain for F2 (c.800C>T, p.A267V) and F3 (c.863C>T, p.T288I), and DNA-binding domain for F4 (c.1074G>T, p.L358F; Figure 2). PBMCs from these patients showed increased STAT1 phosphorylation toward interferon (IFN)-α and IFN-γ as well as delayed STAT1 dephosphorylation in the presence of staurosporine, indicating that they were GOF mutations.

### Family 5

A 9-year old boy (F5.2) was referred for recurrent pneumonia and chronic onychomycosis. Upon inquiry on family history, the boy's father (F5.1) has a history of protracted fungal infection in his childhood. The description of his clinical course could be traced back to a case report by Yuen et al. published in 1986, the first report of pediatric disseminated *T. marneffei* infection in Hong Kong (37). In brief, F5.1 presented with bilateral cervical lymphadenopathy at 10 years old in 1983 and was initially treated with anti-tuberculous drugs. There was progressive enlargement of the cervical lymph nodes complicated by ulceration of the overlying skin and perforation of the hard palate. He was also found to have enlarged mediastinal lymph nodes causing superior vena cava (SVC) obstruction. Tissue from the neck ulcer and axillary lymph node yielded *T. marneffei*. Lymphocyte subset showed profound T-lymphopenia (CD4 93/μl and CD8 138/μl). He was treated with intravenous amphotericin B and oral flucytosine for 3 months with good response. However, 3 years later he presented again with generalized skin lesions, oral ulceration, and recurrence of mediastinal lymphadenopathy. He was treated with amphotericin B for 3 months but shortly after stopping anti-fungal treatment, he had recurrent disease with osteomyelitis involving the left distal radius and ulna, left thumb metacarpus and right tibia (38). Treatment with amphotericin B was resumed but he experienced severe adverse reactions, which necessitated the switch to oral fluconazole. The disease went into remission after 6 months of fluconazole, which he continued taking for a total of 2 years. He had no further disease recurrence and remained largely asymptomatic. His CD4+ and CD8+ T-cells returned to normal, but he was found to have impaired natural killer (NK) cell cytotoxicity (38). He had no medical follow-up since the late 1990s.

F5.1 was 40 years old when he was seen in our clinic together with his son. He had classical features of CMC. He had multiple scars in the neck that corresponded to the history of ulcerated cervical lymphadenopathy. He had mild facial puffiness, and the prominent superficial veins in the neck and upper chest were the result of previous SVC obstruction.

**Figure 3:**
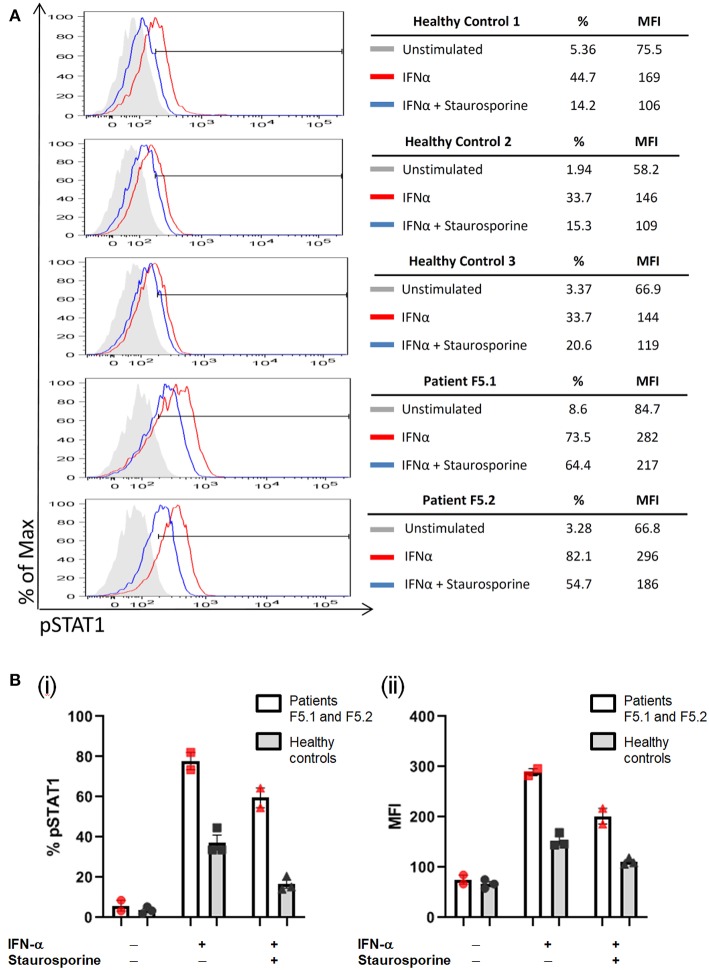
PBMCs were stimulated with IFN-α (40,000 IU/ml) followed by treatment with staurosporine (500 nM) for 30 min, and analyzed for intracellular pSTAT1 expression by gating on CD3+ T-cells. The increase in %pSTAT1^+^ population in stimulated cells relative to unstimulated cells was calculated. **(A)** Representative histograms are shown for Patients F5.1 and F5.2 and 3 healthy controls. **(B)** %pSTAT1^+^ T-cells (i) and mean fluorescent intensity (MFI) (ii) in Patients F5.1 and F5.2 compared with 3 healthy controls. Data expressed as mean ± SEM.

PBMCs obtained from the father and son showed increased STAT1 phosphorylation (pSTAT1) toward IFN-α stimulation (MFI = 282 for F5.1 and 296 for F5.2, vs. 153 ± 8.0 in 3 healthy controls, mean ± SEM), as well as reduced STAT1 dephosphorylation in the presence of staurosporine (Figure 3). A heterozygous missense mutation in the DNA binding domain of the *STAT1* gene (c.1170G>A, p.M390I) was identified by Sanger sequencing (Figure 2). Both patients remained clinically well while on anti-fungal prophylaxis.

### Family 6

F6.2 is the second child of a family belongs to the Karen ethnic group residing in the Thailand-Myanmar border. His parents are third cousins. His elder sister (F6.1) had disseminated *T. marneffei* infection at 5 months, and died of fulminant Salmonella septicemia at 11 months. She was negative for HIV, and immunological investigations including lymphocyte subset, immunoglobulin levels, and dihydrorhodamine reduction (DHR) was unremarkable. WES revealed a novel homozygous frameshift mutation (c.182dupT, p.V61fsX69) in the *IFNGR1* gene, resulting in a premature stop codon upstream to the segment encoding the transmembrane domain (Figure 2Biii). Parents were heterozygous carriers (Figure 2A). F6.2 developed disseminated BCG and Salmonella septicemia at 2 months of age. At 12 months, he was admitted to Chiang Mai University Hospital for high fever and refusal to stand and walk. Blood culture yielded *T. marneffei* and plain X-ray of his legs showed osteolytic lesion in the left distal tibia. He received amphotericin B for 6 weeks with good treatment response. His daily activities returned to normal and he was put on isoniazid, rifampicin and itraconazole prophylaxis.

**Figure 4:**
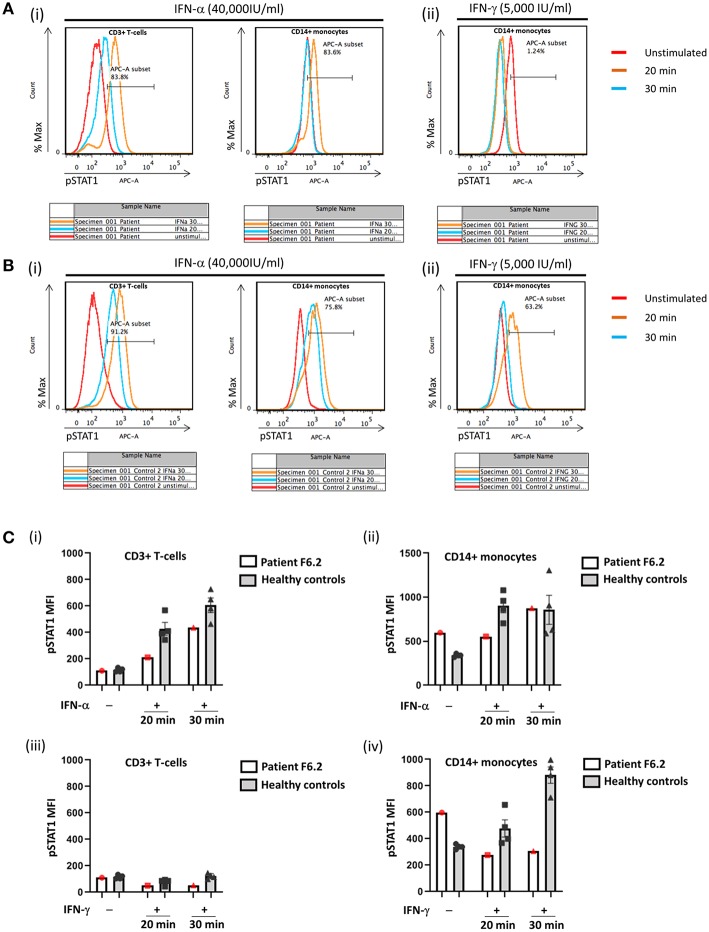
Flow cytometric analysis of STAT1 phosphorylation (pSTAT1) in CD3+ T-cells and CD14+ monocytes in response to IFN-α and IFN-γ. Representative flow plots showing % pSTAT1+ cells in **(A)** Patient F6.2 and **(B)** a healthy control. Peripheral blood mononuclear cells (PBMCs) were stimulated with IFN-α (40,000 IU/ml) or IFN-γ (5,000 IU/ml) for 20 or 30 min. Intracellular phosphorylated STAT1 (pSTAT1) was evaluated by gating on CD3+ and CD14+ cells in PBMCs stimulated with IFN-α **(A**i, **B**i**)** and CD14+ cells in PBMCs stimulated with IFN-γ **(A**ii, **B**ii**)**. **(C)** Mean fluorescent intensity (MFI) of pSTAT1 in Patient F6.2 and 4 healthy controls, data expressed as mean ± SEM.

In view of the family history, Sanger sequencing of the *IFNGR1* gene was performed which showed that F6.2 had the same homozygous frameshift mutation as his deceased sister. PBMCs obtained from F6.2 showed defective STAT1 phosphorylation toward IFN-γ stimulation in CD14+ monocytes compared with 4 healthy controls (Figures 4Aii,Bii,Civ), while STAT1 phosphorylation toward IFN-α stimulation was preserved in CD3+ T-cells (Figures 4Ai,Bi,Ci,ii), implying defective IFN-γ receptor-mediated signaling.

## PIDs in HIV-Negative Children With Talaromycosis and Proposed Diagnostic Algorithm

In addition to the above described patients, 12 cases of pediatric patients with talaromycosis and underlying PIDs were reported in the literature (22–29), and they are summarized in Table 2. Out of these 19 patients, 13 patients (68%) were below the age of 5 years when they had *T. marneffei* infection. 15 patients (79%) had disseminated talaromycosis and two of them had recurrence. Only one patient died of talaromycosis due to multi-organ failure. PIDs diagnosed included X-linked hyper-IgM syndrome (*n* = 10), AD hyper-IgE syndrome (*n* = 3), AD GOF STAT1 disorder (*n* = 4), and autosomal recessive (AR) IFNγR1 deficiency (*n* = 2).

**Table 2:** Primary Immunodeficiencies reported in HIV-negative children with *T. marneffei* infection.

	**Genetic defect**	**Mutation**	**Gender/age, residence**	**Extent of *T. marneffei* infection**	**Treatment and outcome**
F1	CD40L deficiency	g.IVS1+1G>A	M/29 months, China	Disseminated, with recurrent disease	Disseminated disease reated with voriconazole for 4 months with good response, subsequent recurrence as laryngeal involvement also treated with voriconazole with success
Kamchaisatian et al. (24)	CD40L deficiency	Complex mutation in exon 5	M/14 months, Northeastern Thailand	Disseminated	Treated with amphotericin B for 21 days, followed by itraconzole for 10–12 weeks
Kamchaisatian et al. (24)	CD40L deficiency	Not stated	M/1 year, Northern Thailand	Pulmonary disease and lymphadenopathy	Treated with amphotericin B for 21 days, followed by itraconzole for 10–12 weeks
Sripa et al. (25)	CD40L deficiency	Not stated	M/3 years, Thailand	Pulmonary disease	Itraconazole, good response
Liu et al. (27) Du et al. (29)	CD40L deficiency	g.IVS1-3T>G	M/2 years, China	Disseminated	Died of multi-organ failure
Li et al. (23)	CD40L deficiency	Not stated	M/14 months, China	Disseminated	Treated with itraconazole for 2 weeks and improved
Du et al. (29)	CD40L deficiency	g.IVS3+1G>A	M/35 months, China	Disseminated	Responded well to anti-fungal therapy
Du et al. (29)	CD40L deficiency	g.IVS1-1G>A	M/27 months, China	Disseminated	Lost to follow-up
Du et al. (29)	CD40L deficiency	g.IVS4+1G>C	M/3 years, China	Disseminated	Responded well to anti-fungal therapy
Du et al. (29)	CD40L deficiency	Large fragment deletion including exon 4 and exon 5	M/13 years, China	Disseminated	Responded well to anti-fungal therapy
Ma et al. (26)	AD Hyper-IgE syndrome (STAT3)	Not stated	M/30 years, Hong Kong, China	Pulmonary (co-infection with *Stenotrophomonas maltophilia*)	Treated with amphotericin B, died of respiratory failure due to rapid disease progression
Lee et al. (22)	AD Hyper-IgE syndrome (STAT3)	p.D374G	F/12 months, China	Disseminated	Treated with itraconazole with good response
Fan et al. (28)	AD Hyper-IgE syndrome (STAT3)	p.K531N	M/13 years, China	Disseminated	Amphotericin B and voriconazole for 2 weeks, followed by itraconzole for 2 months
F2	AD gain-of-function STAT1 disorder	p.A267V	M/15 years, Hong Kong, China	Disseminated	Treated with amphotericin B for 6 weeks with good response, followed by itraconazole prophylaxis
F3	AD gain-of-function STAT1 disorder	p.T288I	F/7 years, Hong Kong, China	Pulmonary (co-infection with cytomegalovirus)	Treated with amphotericin B for 6 weeks with good response, followed by itraconazole prophylaxis
F4	AD gain-of-function STAT1 disorder	p.L358F	F/5 years, Hong Kong, China	Cervical lymphadenopathy (co-infection with *Mycobacterium tuberculosis*)	Treated with itraconazole and anti-tuberculous treatment with good response
F5	AD gain-of-function STAT1 disorder	p.M390I	M/10 years, Hong Kong, China	Disseminated with multiple recurrences	Protracted courses of anti-fungal therapy with eventual clearance
F6.1	AR IFNGR1 deficiency	Homozygous p.V61fsX69	F/5 months, Northern Thailand	Disseminated	Treated with amphotericin B for 6 weeks with good response, followed by itraconazole prophylaxis
F6.2	AR IFNGR1 deficiency	Homozygous p.V61fsX69	M/12 months, Northern Thailand	Disseminated	Treated with amphotericin B for 6 weeks with good response, followed by itraconazole prophylaxis

**Figure 5:**
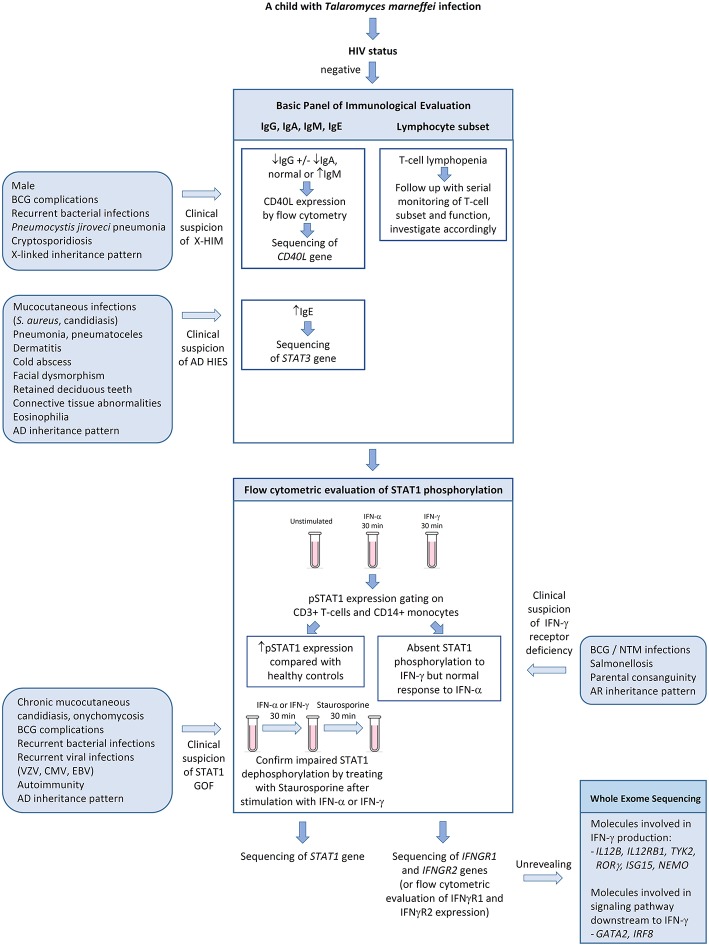
Algorithm for immunological evaluation of pediatric patientswith disseminated *Talaromyces marneffei* infection. AD, autosomal dominant; BCG, Bacille Calmette-Guerin; CMV, cytomegalovirus; EBV, Epstein-Barr virus; GOF, gain-of-function; HIES, hyper-IgE syndrome; NTM, non-tuberculous mycobacteria; pSTAT1, phosphorylated STAT1; VZV, varicella zoster virus; X-HIM, X-linked hyper-IgM syndrome.

We propose a diagnostic algorithm targeting at the above PIDs for immunological evaluation of HIV-negative children with talaromycosis in whom secondary causes of immunosuppression are excluded (Figure 5). History taking and physical examination should focus on identifying past history or concurrent opportunistic infections including BCG complications and non-tuberculous mycobacteria infections, salmonellosis, *Pneumocystis jiroveci* pneumonia, cryptosporidiosis, severe human herpes virus infections (e.g., varicella zoster virus, cytomegalovirus, and Epstein Barr virus), chronic mucocutaneous candidiasis, onychomycosis, and other invasive fungal infections such as aspergillosis. Recurrent sinopulmonary infections are common in CD40L deficiency and AD GOF STAT1 disorder. Coarse facies, high-arched palate, retention of deciduous teeth, scoliosis, cold abscesses and pneumatoceles are suggestive of AD hyper-IgE syndrome. Detailed family history on recurrent infections, early infant deaths and parental consanguinity should be sought. A basic panel of immunological investigations including immunoglobulin pattern (IgG, IgA, IgM, and IgE) and lymphocyte subset should be performed. Low IgG, low IgA, and normal or high IgM in a male patient raises suspicion for CD40L deficiency and one should proceed with flow cytometric evaluation of CD40L expression. The presence of pneumoatoceles, eosinophilia, and elevated IgE, in the presence of somatic features of AD hyper-IgE syndrome, should prompt genetic confirmation of *STAT3* gene mutation. Defects in interferon-mediated JAK-STAT signaling are evaluated by STAT1 phosphorylation studies by flow cytometry. STAT1 hyperphosphorylation in response to IFN-α or IFN-γ and delayed dephosphorylation in the presence of staurosporine is diagnostic for GOF STAT1 disorder, while absent STAT1 phosphorylation in response to IFN-γ but normal response to IFN-α is suggestive of IFN-γ receptor deficiency. Although endemic mycoses have not been reported in patients with chronic granulomatous disease (CGD), it is reasonable to include nitroblue tetrazolium test (NBT) or dihydrorhodamine test (DHR) as a screening strategy for invasive fungal infections. In older children or teenagers who are otherwise healthy, autoantibodies against IFN-γ should be measured.

## Discussion

The mechanism of immune response toward penicilliosis is poorly understood. Human penicilliosis is believed to be initiated by inhalation of conidia which are subsequently phagocytosed by alveolar macrophages. They survive in the intracellular environment of macrophages and develop into the yeast phase. Once established within the macrophages, *T. marneffei* readily disseminates throughout the body causing systemic infection when host immune response is suppressed. Clinically, only the yeast form is found in tissues and peripheral blood (3, 39). Only a few studies on cell mediated immune response toward *T. marneffei* were available in the literature. *T. marneffei* infection is fatal in nude mice or T-cell depleted mice, indicating the importance of T-cell response in the immune defense against *T. marneffei* (40, 41). Fungicidal activity of *T. marneffei* yeast by human and murine macrophages could be enhanced by IFNγ via stimulation of L-arginine-dependent nitric oxide pathway (42). In mice infected by *T. marneffei*, a Th1-polarized pattern of cytokines (IFN-γ and IL-12) was observed in the spleen, and systemic *T. marneffei* infection was invariably fatal in IFN-γ-knockout mice (43). The importance of IFN-γ in host defense against T. marneffei is best illustrated by the association of high-titer anti-IFNγ autoantibody with disseminated non-tuberculous mycobacteria (NTM) infection, talaromycosis, histoplasmosis, cryptococcosis, melioidosis, non-typhoidal salmonellosis, and severe varicella zoster virus infections in adults without HIV infection (15–20). These infections are typical of advanced AIDS despite the fact that these patients had essentially normal numbers of CD4+ T cells and other lymphocytes (17). F6.1 and F6.2 were the first cases of invasive mycoses reported in AR IFNγR1 deficiency. Susceptibility to candidiasis and filamentous fungi has not been described in patients with AR IFNγR1 deficiency; instead, histoplasmosis (44) and coccidioidomycosis (45) were reported in patients with AD partial IFN-γR1 deficiency residing in endemic regions in the United States. Both patients had disseminated mycoses with lymphadenopathy, pulmonary and skeletal involvement necessitating surgical intervention, and recurrent/refractory disease course requiring prolonged intensive anti-fungal treatment, as well as concomitant NTM infections. Both patients received IFN-γ as an adjunctive treatment that led to clearance of infections. Taken together, it is most likely that IFN-γ plays a critical role in host immunity against dimorphic fungi.

The current understanding about inborn errors of immunity predisposing to talaromycosis is limited. The geographical regions with the highest incidence of *T. marneffei* infections are relatively less developed in terms of PID specialist service. It is likely that many HIV-negative children with talaromycosis have not received thorough immunological investigations, and hence the proportion of such cases with underlying PIDs is unknown. Through our APID Network and literature search, the types of PIDs that have been documented in children with *T. marneffei* infection include CD40L deficiency, AD hyper-IgE syndrome, GOF STAT1 disorder and AD IFN-γR1 deficiency. These PIDs have also been identified in other endemic mycoses including histoplasmosis (44, 46–61), coccidioidomycosis (45, 53, 61–63) and paracoccidioidomycosis (64). Such dimorphic fungi have also caused disseminated disease in patients with IL12Rβ1 deficiency (65–69). In addition, disseminated histoplasmosis was reported in patients with GATA2 deficiency (52, 70) and NEMO deficiency (52), mainly in adults. The susceptibility to endemic mycoses in CD40L, NEMO, IL12Rβ1 and IFN-γR1 deficiencies highlights the critical role of IL-12/IFN-γ crosstalk in macrophage activation and killing of these dimorphic fungi. On the other hand, impaired Th17 response in GOF STAT1 defect and AD hyper-IgE syndrome leads to CMC and invasive fungal infections caused by filamentous and dimorphic fungal pathogens. In fact, IL12Rβ1, NEMO, CD40L, and IFNγR1 deficiencies also result in impaired Th17 generation from naïve T-cells which likely contribute to susceptibility to fungal infection (71). There is a gap of knowledge in Th17-mediated cellular response against *T. marneffei* infection and mechanistic studies are required. Endemic mycoses have not been reported in patients with CGD (72), so it appears that defective oxidative burst *per se* is not sufficient to cause an increased risk to dimorphic fungi, suggesting that other mechanisms of phagosomal killing may compensate for the lack of NADPH oxidase activity in eliminating these pathogens in the cytosolic compartment.

An algorithmic approach in history taking, targeted physical examination and stepwise immunological investigations will be helpful to assist clinicians in recognizing and diagnosing PIDs in patients with talaromycosis who are HIV-negative. Flow cytometric evaluation of CD40L expression and STAT1 phosphorylation is simple and rapid, with a turnaround time of less than 1 day to obtain a result that can provide important diagnostic information that guides treatment and genetic confirmation. STAT1 phosphorylation serve as a functional test of cellular responses mediated by type I (IFN-α/β) and type II (IFN-γ) IFNs. Flow cytometry-based STAT1 functional study has been described by Mizoguchi et al. (73) and Bitar et al. (74) as a rapid screening method to facilitate the diagnosis of CMC caused by GOF STAT1, which is characterized by STAT1 hyperphosphorylation in response to IFN-α or IFN-γ stimulation, and delayed dephosphorylation in the presence of staurosporine. Absence of STAT1 phosphorylation in response to IFN-γ but present in IFN-α stimulation suggests IFN-γ receptor deficiency. Impaired or absent STAT1 phosphorylation in response to both IFN-α and IFN-γ suggest loss-of-function STAT1 defect, and possibly JAK1 defect (75). Such functional defects, if present, should be confirmed by gene sequencing. If these tests are unrevealing, genetic defects of other molecules involved in IFN-γ production (IL12B, IL12Rβ1, TYK2, RORγ, ISG15, NEMO) or signaling pathway downstream to IFN-γ (GATA2, IRF8) should be considered (76), given the central importance of IFN-γ in host defense against *T. marneffei*. Considering the broad diagnostic possibilities, it would be reasonable to proceed with whole exome sequencing to identify the causative gene mutations. It is likely that increased awareness and improved diagnostics will unveil more PIDs underlying HIV-negative talaromycosis and other endemic mycoses, which will in turn advance our understanding about human immune response against this distinctive group of pathogenic fungi.

## Data Availability

All datasets generated for this study are included in the manuscript/Supplementary Files.

## Ethics Statement

The studies involving human participants were reviewed and approved by Institutional Review Board of The University of Hong Kong/Hospital Authority Hong Kong West Cluster. Written informed consent to participate in this study was provided by the participants' legal guardian/next of kin. Written informed consent was obtained from the individual(s), and minor(s)' legal guardian/next of kin, for the publication of any potentially identifiable images or data included in this article.

## Author Contributions

PL wrote the article and supervised STAT1 functional studies performed in patients F5.1, F5.2 and F6.2. JY, K-WC, and WY performed genetic diagnostics studies for all patients reported in this article. HMao designed the flow cytometric protocol for STAT1 phosphorylation studies, and performed the functional studies for patients F2, F3, and F4. HMao provided clinical information of patient F1. ML and MT provided clinical information of Family 6. XZ provided flow cytometric data on patient F1. HMa, L-CP, and LK performed STAT1 functional studies. Y-LL provided the conceptual framework and edited the manuscript.

### Conflict of Interest Statement

The authors declare that the research was conducted in the absence of any commercial or financial relationships that could be construed as a potential conflict of interest.

## References

[1] Deng Z Ribas JL Gibson DW Connor DH. Infections caused by *Penicillium marneffei* in China and Southeast Asia: review of eighteen published cases and report of four more Chinese cases. Rev Infect Dis. (1988) 10:640–52. 10.1093/clinids/10.3.640 3293165

[2] Li PC Yeoh EK. Current epidemiological trends of HIV infection in Asia. AIDS Clin Rev. (1992) 1–23. 1606054

[3] Supparatpinyo K Khamwan C Baosoung V Nelson KE Sirisanthana T. Disseminated *Penicillium marneffei* infection in southeast Asia. Lancet. (1994) 344:110–3. 10.1016/S0140-6736(94)91287-4 7912350

[4] Kaldor JM Sittitrai W John TJ Kitamura T. The emerging epidemic of HIV infection and AIDS in Asia and the Pacific. AIDS. (1994) 8:S1–2. 7857551

[5] Duong TA. Infection due to *Penicillium marneffei*, an emerging pathogen: review of 155 reported cases. Clin Infect Dis. (1996) 23:125–30. 10.1093/clinids/23.1.125 8816141

[6] World Health Organization WHO Case Definitions of HIV for Surveillance and Revised Clinical Staging and Immunological Classification of HIV-Related Disease in Adults and Children. WHO Press (2007).

[7] Chierakul W Rajanuwong A Wuthiekanun V Teerawattanasook N Gasiprong M Simpson A. The changing pattern of bloodstream infections associated with the rise in HIV prevalence in northeastern Thailand. Trans R Soc Trop Med Hyg. (2004) 98:678–86. 10.1016/j.trstmh.2004.01.011 15363648

[8] Nga TV Parry CM Le T Lan NP Diep TS Campbell JI. The decline of typhoid and the rise of non-typhoid salmonellae and fungal infections in a changing HIV landscape: bloodstream infection trends over 15 years in southern Vietnam. Trans R Soc Trop Med Hyg. (2012) 106:26–34. 10.1016/j.trstmh.2011.10.004 22137537

[9] Vanittanakom N Cooper CR Jr Fisher MC Sirisanthana T. *Penicillium marneffei* infection and recent advances in the epidemiology and molecular biology aspects. Clin Microbiol Rev. (2006) 19:95–110. 10.1128/CMR.19.1.95-110.2006 16418525 PMC1360277

[10] Le T Wolbers M Chi NH Quang VM Chinh NT Lan NP. Epidemiology, seasonality, and predictors of outcome of AIDS-associated *Penicillium marneffei* infection in Ho Chi Minh City, Viet Nam. Clin Infect Dis. (2011) 52:945–52. 10.1093/cid/cir028 21427403 PMC3106230

[11] Masannat FY Ampel NM. Coccidioidomycosis in patients with HIV-1 infection in the era of potent antiretroviral therapy. Clin Infect Dis. (2010) 50:1–7. 10.1086/648719 19995218

[12] Brown J Benedict K Park BJ Thompson GR III. Coccidioidomycosis: epidemiology. Clin Epidemiol. (2013) 5:185–97. 10.2147/CLEP.S34434 23843703 PMC3702223

[13] Wong SS Wong KH Hui WT Lee SS Lo JY Cao L. Differences in clinical and laboratory diagnostic characteristics of *Penicilliosis marneffei* in human immunodeficiency virus (HIV)- and non-HIV-infected patients. J Clin Microbiol. (2001) 39:4535–40. 10.1128/JCM.39.12.4535-4540.2001 11724878 PMC88582

[14] Chan JF Lau SK Yuen KY Woo PC. *Talaromyces (Penicillium) marneffei* infection in non-HIV-infected patients. Emerg Microbes Infect. (2016) 5:e19. 10.1038/emi.2016.18 26956447 PMC4820671

[15] Tang BS Chan JF Chen M Tsang OT Mok MY Lai RW. Disseminated penicilliosis, recurrent bacteremic nontyphoidal salmonellosis, and burkholderiosis associated with acquired immunodeficiency due to autoantibody against gamma interferon. Clin Vaccine Immunol. (2010) 17:1132–8. 10.1128/CVI.00053-10 20445006 PMC2897261

[16] Kampitak T Suwanpimolkul G Browne S Suankratay C. Anti-interferon-γ autoantibody and opportunistic infections: case series and review of the literature. Infection. (2011) 39:65–71. 10.1007/s15010-010-0067-3 21128092

[17] Browne SK Burbelo PD Chetchotisakd P Suputtamongkol Y Kiertiburanakul S Shaw PA. Adult-onset immunodeficiency in Thailand and Taiwan. N Engl J Med. (2012) 367:725–34. 10.1056/NEJMoa1111160 22913682 PMC4190026

[18] Wongkulab P Wipasa J Chaiwarith R Supparatpinyo K. Autoantibody to interferon-gamma associated with adult-onset immunodeficiency in non-HIV individuals in Northern Thailand. PLoS ONE. (2013) 8:e76371. 10.1371/journal.pone.0076371 24086734 PMC3785451

[19] Chan JF Trendell-Smith NJ Chan JC Hung IF Tang BS Cheng VC. Reactive and infective dermatoses associated with adult-onset immunodeficiency due to anti-interferon-gamma autoantibody: sweet's syndrome and beyond. Dermatology. (2013) 226:157–66. 10.1159/000347112 23652167

[20] Chi CY Lin CH Ho MW Ding JY Huang WC Shih HP. Clinical manifestations, course, and outcome of patients with neutralizing anti-interferon-γ autoantibodies and disseminated nontuberculous mycobacterial infections. Medicine. (2016) 95:e3927. 10.1097/MD.0000000000003927 27336882 PMC4998320

[21] Lee PP Chan KW Lee TL Ho MH Chen XY Li CH. Penicilliosis in children without HIV infection – are they immunodeficient? Clin Infect Dis. (2012) 54:e8–19 10.1093/cid/cir754 22065867

[22] Lee PP Mao H Yang W Chan KW Ho MH Lee TL. *Penicillium marneffei* infection and impaired IFN-γ immunity in humans with autosomal- dominant gain-of-phosphorylation STAT1 mutations. J Allergy Clin Immunol. (2014) 133:894–6.e5. 10.1016/j.jaci.2013.08.051 24188975

[23] Li L Li J Zheng R Zheng Y Wang W Clinical analysis of 3 HIV negative children with penicilliosis. Jiangxi Med J. (2018) 53:986–9. 10.3969/j.issn.1006-2238.2018.9.028

[24] Kamchaisatian W Kosalaraksa P Benjaponpitak S Hongeng S Direkwattanachai C Lumbiganon P Penicilliois in patients with X-linked hyperimmunoglobulin M syndrome (XHIGM), case reports from Thailand. J Allergy Clin Immunol. (2006) 117:S282 10.1016/j.jaci.2005.12.1166

[25] Sripa C Mitchai J Thongsri W Sripa B. Diagnostic cytology and morphometry of *Penicillium marneffei* in the sputum of a hypogammaglobulinemia with hyper-IgM patient. J Med Assoc Thai. (2010) 93:S69–72. 21299093

[26] Ma BH Ng CS Lam R Wan S Wan IY Lee TW. Recurrent hemoptysis with *Penicillium marneffei* and *Stenotrophomonas maltophilia* in Job's syndrome. Can Respir J. (2009) 16:e50–2. 10.1155/2009/586919 19707602 PMC2734441

[27] Liu D Zhong LL Li Y Chen M. [Recurrent fever, hepatosplenomegaly and eosinophilia in a boy]. Zhongguo Dang Dai Er Ke Za Zhi. (2016) 18:1145–9. 10.7499/j.issn.1008-8830.2016.11.018 27817782 PMC7389858

[28] Fan H Huang L Yang D Lin Y Lu G Xie Y. Pediatric hyperimmunoglobulin E syndrome: a case series of 4 children in China. Medicine. (2018) 97:e0215. 10.1097/MD.0000000000010215 29620631 PMC5902260

[29] Du X Tang W Chen X Zeng T Wang Y Chen Z. Clinical, genetic, and immunological characteristics of 40 Chinese patients with CD40 ligand deficiency. Scand J Immunol. (2019). 10.1111/sji.12798. [Epub ahead of print]. 31179555

[30] Zhou L Liu Y Wang H [Chyloascites in a HIV-negative child with *T. marneffei* infection]. J Clin Pediatr. (2015) 33:914–5. 10.3969/j.issn.1000-3606.2015.10.019

[31] Zeng W Qiu Y Lu D Zhang J Zhong X Liu G. A Retrospective analysis of 7 human immunodeficiency virus-negative infants infected by *Penicillium marneffei*. Medicine (Baltimore). (2015) 94:e1439. 10.1097/MD.0000000000001439 26313802 PMC4602911

[32] Li Y Lin Z Shi X Mo L Li W Mo W. Retrospective analysis of 15 cases of *Penicillium marneffei* infection in HIV-positive and HIV-negative patients. Microb Pathog. (2017) 105:321–5. 10.1016/j.micpath.2017.01.026 28104384

[33] Lei M Yu U Zhang N Deng J. An HIV-negative infant with systemic *Talaromyces marneffei* infection. Int J Infect Dis. (2018) 77:3–4. 10.1016/j.ijid.2018.06.003 29906603

[34] Han XJ Su DH Yi JY Zou YW Shi YL. A Literature review of blood-disseminated *P. marneffei* Infection and a case study of this infection in an HIV-Negative Child with Comorbid Eosinophilia. Mycopathologia. (2019) 184:129–39. 10.1007/s11046-018-0255-8 29524085

[35] Lee PP Lau YL. Improving care, education, and research: the Asian primary immunodeficiency network. Ann N Y Acad Sci. (2011) 1238:33–41. 10.1111/j.1749-6632.2011.06225.x 22129051

[36] An Y Xiao J Jiang L Yang X Yu J Zhao X. Clinical and molecular characterization of X-linked hyper-IgM syndrome patients in China. Scand J Immunol. (2010) 72:50–6. 10.1111/j.1365-3083.2010.02406.x 20591076

[37] Yuen WC Chan YF Loke SL Seto WH Poon GP Wong KK. Chronic lymphadenopathy caused by *Penicillium marneffei*: a condition mimicking tuberculous lymphadenopathy. Br J Surg. (1986) 73:1007–8. 10.1002/bjs.1800731224 3790946

[38] Lau GKK Lau CR Kumana KL Wong KY Yuen PY Chau FL Disseminated *Penicillium marneffei* infection responding to treatment with oral fluconazole. J Hong Kong Med Assoc. (1992) 44:176–80.

[39] Cooper CR Vanittanakom N. Insights into the pathogenicity of *Penicillium marneffei*. Future Microbiol. (2008) 3:43–55. 10.2217/17460913.3.1.43 18230033

[40] Kudeken N Kawakami K Kusano N Saito A. Cell-mediated immunity in host resistance against infection caused by *Penicillium marneffei*. J Med Vet Mycol. (1996) 34:371–8. 10.1080/02681219680000671 8971625

[41] Kudeken N Kawakami K Saito A. CD4+ T cell-mediated fatal hyperinflammatory reactions in mice infected with *Penicillium marneffei*. Clin Exp Immunol. (1997) 107:468–73. 10.1046/j.1365-2249.1997.d01-945.x 9067519

[42] Kudeken N Kawakami K Saito A. Different susceptibilities of yeasts and conidia of *Penicillium marneffei* to nitric oxide (NO)-mediated fungicidal activity of murine macrophages. Clin Exp Immunol. (1998) 112:287–93. 10.1046/j.1365-2249.1998.00565.x 9649193 PMC1904956

[43] Sisto F Miluzio A Leopardi O Mirra M Boelaert JR Taramelli D. Differential cytokine pattern in the spleens and livers of BALB/c mice infected with *Penicillium marneffei*: protective role of gamma interferon. Infect Immun. (2003) 71:465–73. 10.1128/IAI.71.1.465-473.2003 12496197 PMC143270

[44] Zerbe CS Holland SM. Disseminated histoplasmosis in persons with interferon-gamma receptor 1 deficiency. Clin Infect Dis. (2005) 41:e38–41. 10.1086/432120 16028145

[45] Vinh DC Masannat F Dzioba RB Galgiani JN Holland SM. Refractory disseminated coccidioidomycosis and mycobacteriosis in interferon-gamma receptor 1 deficiency. Clin Infect Dis. (2009) 49:e62–5. 10.1086/605532 19681704 PMC2730428

[46] Tu RK Peters ME Gourley GR Hong R. Esophageal histoplasmosis in a child with immunodeficiency with hyper-IgM. AJR Am J Roentgenol. (1991) 157:381–2. 10.2214/ajr.157.2.1853826 1853826

[47] Hostoffer RW Berger M Clark HT Schreiber JR. Disseminated *Histoplasma capsulatum* in a patient with hyper IgM immunodeficiency. Pediatrics. (1994) 94:234–6. 8036080

[48] Yilmaz GG Yilmaz E Coşkun M Karpuzoglu G Gelen T Yegin O. Cutaneous histoplasmosis in a child with hyper-IgM. Pediatr Dermatol. (1995) 12:235–8. 10.1111/j.1525-1470.1995.tb00166.x 7501554

[49] Danielian S Oleastro M Eva Rivas M Cantisano C Zelazko M. Clinical follow- up of 11 Argentinian CD40L-deficient patients with 7 unique mutations including the so-called milder mutants. J Clin Immunol. (2007) 27:455–9. 10.1007/s10875-007-9089-8 17351759

[50] Dahl K Eggebeen A Hyper IgM, histoplasmosis and MAS. J Clin Immunol. (2012) 32:355.

[51] Pedroza LA Guerrero N Stray-Pedersen A Tafur C Macias R Muñoz G. First case of CD40LG deficiency in Ecuador, diagnosed after whole exome sequencing in a patient with severe cutaneous histoplasmosis. Front Pediatr. (2017) 5:17. 10.3389/fped.2017.00017 28239602 PMC5300990

[52] Lovell JP Foruraghi L Freeman AF Uzel G Zerbe CS Su H. Persistent nodal histoplasmosis in nuclear factor kappa B essential modulator deficiency: report of a case and review of infection in primary immunodeficiencies. J Allergy Clin Immunol. (2016) 138:903–5. 10.1016/j.jaci.2016.02.040 27266944 PMC5391257

[53] Sampaio EP Hsu AP Pechacek J Bax HI Dias DL Paulson ML. Signal transducer and activator of transcription 1 (STAT1) gain-of-function mutations and disseminated coccidioidomycosis and histoplasmosis. J Allergy Clin Immunol. (2013) 131:1624–34. 10.1016/j.jaci.2013.01.052 23541320 PMC3746066

[54] Alberti-Flor JJ Granda A. Ileocecal histoplasmosis mimicking Crohn's disease in a patient with Job's syndrome. Digestion. (1986) 33:176–80. 10.1159/000199290 3949095

[55] Cappell MS Manzione NC. Recurrent colonic histoplasmosis after standard therapy with amphotericin B in a patient with Job's syndrome. Am J Gastroenterol. (1991) 86:119–20. 1986542

[56] Desai K Huston DP Harriman GR. Previously undiagnosed hyper-IgE syndrome in an adult with multiple systemic fungal infections. J Allergy Clin Immunol. (1996) 98:1123–4. 10.1016/S0091-6749(96)80202-8 8977516

[57] Steiner SJ Kleiman MB Corkins MR Christenson JC Wheat LJ. Ileocecal histoplasmosis simulating Crohn disease in a patient with hyperimmunoglobulin E syndrome. Pediatr Infect Dis J. (2009) 28:744–6. 10.1097/INF.0b013e31819b65e0 19633521

[58] Robinson WS Arnold SR Michael CF Vickery JD Schoumacher RA Pivnick EK. Case report of a young child with disseminated histoplasmosis and review of hyper immunoglobulin e syndrome (HIES). Clin Mol Allergy. (2011) 9:14. 10.1186/1476-7961-9-14 22126402 PMC3248830

[59] Rana C Krishnani N Kumari N Shastri C Poddar U. Rectal histoplasmosis in Job's syndrome. Indian J Gastroenterol. (2013) 32:64–5. 10.1007/s12664-012-0275-0 23151895

[60] Jiao J Horner CC Kau AL Terminal ileum perforation in a patient with hyper-IgE syndrome. Ann Allergy Asthma Immunol. (2014) 113:S61.

[61] Odio CD Milligan KL McGowan K Rudman Spergel AK Bishop R Boris L. Endemic mycoses in patients with STAT3-mutated hyper-IgE (Job) syndrome. J Allergy Clin Immunol. (2015) 136:1411–3.e1–2. 10.1016/j.jaci.2015.07.003 26292779 PMC4641001

[62] Stanga SD Dajud MV. Visual changes in a 4-year-old. Clin Pediatr. (2008) 47:959–61. 10.1177/0009922808319788 18987292

[63] Powers AE Bender JM Kumánovics A Ampofo K Augustine N Pavia AT. *Coccidioides immitis* meningitis in a patient with hyperimmunoglobulin E syndrome due to a novel mutation in signal transducer and activator of transcription. Pediatr Infect Dis J. (2009) 28:664–6. 10.1097/INF.0b013e31819866ec 19483664

[64] Cabral-Marques O Schimke LF Pereira PV Falcai A de Oliveira JB Hackett MJ. Expanding the clinical and genetic spectrum of human CD40L deficiency: the occurrence of paracoccidioidomycosisand other unusual infections in Brazilian patients. J Clin Immunol. (2012) 32:212–20. 10.1007/s10875-011-9623-6 22193914

[65] Moraes-Vasconcelos DD Grumach AS Yamaguti A Andrade ME Fieschi C de Beaucoudrey L. *Paracoccidioides brasiliensis* disseminated disease in a patient with inherited deficiency in the beta1 subunit of the interleukin (IL)-12/IL-23 receptor. Clin Infect Dis. (2005) 41:e31–7. 10.1086/432119 16028144

[66] de Beaucoudrey L Puel A Filipe-Santos O Cobat A Ghandil P Chrabieh M. Mutations in STAT3 and IL12RB1 impair the development of human IL-17-producing T cells. J Exp Med. (2008) 205:1543–50. 10.1084/jem.20080321 18591412 PMC2442631

[67] Hwangpo TA Harriw WT Atkinson P Cassady K Kankirawatana S IL-12 receptor defect predisposes to histoplasmosis. Ann Allergy Asthma Immunol. (2012) 109:A80.

[68] Falcão ACAM Marques PTL Santos AR Oliveira JB Disseminated histoplasmosis caused by IL12RB1 gene mutations in two Brazilian siblings. J Clin Immunol. (2012) 32:405.

[69] Louvain de Souza T de Souza Campos Fernandes RC Azevedo da Silva J Gomes Alves Júnior V Gomes Coelho A Souza Faria AC. Microbial disease spectrum linked to a novel IL-12Rβ1 N-terminal signal peptide stop-gain homozygous mutation with paradoxical receptor cell-surface expression. Front Microbiol. (2017) 8:616. 10.3389/fmicb.2017.00616 28450854 PMC5389975

[70] Spinner MA Sanchez LA Hsu AP Shaw PA Zerbe CS Calvo KR. GATA2 deficiency: a protean disorder of hematopoiesis, lymphatics, and immunity. Blood. (2014) 123:809–21. 10.1182/blood-2013-07-515528 24227816 PMC3916876

[71] Ma CS Wong N Rao G Nguyen A Avery DT Payne K. Unique and shared signaling pathways cooperate to regulate the differentiation of human CD4+ T cells into distinct effector subsets. J Exp Med. (2016) 213:1589–608. 10.1084/jem.20151467 27401342 PMC4986526

[72] Holland SM. Chronic granulomatous disease. Hematol Oncol Clin North Am. (2013) 27:89–99. 10.1016/j.hoc.2012.11.002 23351990 PMC3558921

[73] Mizoguchi Y Tsumura M Okada S Hirata O Minegishi S Imai K. Simple diagnosis of STAT1 gain-of-function alleles in patients with chronic mucocutaneous candidiasis. J Leukoc Biol. (2014) 95:667–76. 10.1189/jlb.0513250 24343863 PMC3958742

[74] Bitar M Boldt A Binder S Borte M Kentouche K Borte S. Flow cytometric measurement of STAT1 and STAT3 phosphorylation in CD4_+_ and CD8_+_ T cells-clinical applications in primary immunodeficiency diagnostics. J Allergy Clin Immunol. (2017) 140:1439–41.e9. 10.1016/j.jaci.2017.05.017 28601682

[75] Eletto D Burns SO Angulo I Plagnol V Gilmour KC Henriquez F. Biallelic JAK1 mutations in immunodeficient patient with mycobacterial infection. Nat Commun. (2016) 7:13992. 10.1038/ncomms13992 28008925 PMC5196432

[76] Rosain J Kong XF Martinez-Barricarte R Oleaga-Quintas C Ramirez-Alejo N Markle J. Mendelian susceptibility to mycobacterial disease: 2014-2018 update. Immunol Cell Biol. (2019) 97:360–7. 10.1111/imcb.12210 30264912 PMC6438774

