# Supplementary material for: Application of Flow Cytometry in the Diagnostics Pipeline of Primary Immunodeficiencies Underlying Disseminated Talaromyces marneffei Infection in HIV-Negative Children
Source: Front Immunol. 2019 Sep 13;10:2189. doi: 10.3389/fimmu.2019.02189 (PMC6753679; doi:10.3389/fimmu.2019.02189)
Supplement: Supplementary file 1 [file Table_1.DOCX]

Supplementary Table 1 Primer sequences for CD40LG, IFNGR1 and STAT1 genes

XHIM (CD40L)

NC_000023, NM_000074

| Primer Name | Sequence 5’🡪 3’ | Bases |
| --- | --- | --- |
| TNFSF5-P1F | CGTTTTTGCTGGGAGAGAAGAC | 22 |
| TNFSF5-E1RA | CCTATGAATTAGTAAGGACCCCAA | 24 |
| TNFSF5-E2FA | CTGTATTCTCCTTCCGAATGAC | 22 |
| TNFSF5-E2RA | TGTCAGTTTCCCGATCTAGCA | 21 |
| TNFSF5-E3FB | CATGGACTCCCAATTGGCATGA | 22 |
| TNFSF5-E3RB | CAGGGACCAGAGAAGGTGTCA | 21 |
| TNFSF5-E4FA | ACCAGATAGTTTTGTGGGCAGT | 22 |
| TNFSF5-E4RA | CTTTCCCCTTGGGTGGCTAC | 20 |
| TNFSF5-E5FA | CTCTGCTTCACCTCACCACAA | 21 |
| TNFSF5-E5RA | CCACAGCCTGCAAGGTGACA | 23 |

IFNGR1

NC_000006, NM_000416

| Primer Name | Sequence 5’🡪 3’ | Bases |
| --- | --- | --- |
| IFNGR1-E1F-PROF | GCTGTGGGAATCTGCACAAACC | 22 |
| IFNGR1-E1R | CTAGGGCGACCTCGGAGAAG | 20 |
| IFNGR1-E2F | CATATCTGGGCAATGTGGCATC | 22 |
| IFNGR1-E2R | GCCACGTGGGAAGGCTGATG | 20 |
| IFNGR1-E3F | CCATCCTATTCTTAGCTCTGCT | 22 |
| IFNGR1-E3R | ATGCTCAACCTGTACTGACTCT | 22 |
| IFNGR1-E4F | TGGTCCTGCTTTAGAACAACCA | 22 |
| IFNGR1-E4R | TCACATGGTCAGTGTTAGTGAC | 22 |
| IFNGR1-E5F | TGCATAGTATCGTGCTGTGTTG | 22 |
| IFNGR1-E5F-SEQ | TTTCAGATTAAAAGAAGCTGTGCA | 24 |
| IFNGR1-E5R | TCTCTAAGGAATGGAACTAATGC | 23 |
| IFNGR1-E6F | CTTCTGCTTCCTTCAGTGTTCT | 22 |
| IFNGR1-E6R | GACTGATTGATGGCAGGTGACA | 22 |
| IFNGR1-E7F | AATGTGCCATTTGGTGGTCCAT | 22 |
| IFNGR1-E7R-cDNAR | CACTAAGTCACTCCATTTGGTTG | 23 |

STAT1

NC_000002, NM_007315

| Primer Name | Sequence 5’🡪 3’ | Bases |
| --- | --- | --- |
| STAT1-P1F | GGACTGGGCTGCAGCTCAC | 19 |
| STAT1-E1R | CGCGTTCTTCCTCTGGGATC | 20 |
| STAT1-E2F | CAGTCGTGCTCTGGCAGTGA | 20 |
| STAT1-E2R | GAAGACATTAAGCCCTTCCATC | 22 |
| STAT1-E3F | CCTGGGACAAACTATGTTTTGG | 22 |
| STAT1-E3R | AATTCCAGTGGCCATTGATGGA | 22 |
| STAT1-E4F | CTGCTGCTAAAAGCAATGTCAG | 22 |
| STAT1-E4R | GGAGTTAGTCTCTATTACTTCTC | 23 |
| STAT1-E5F | GAGATGAGGTTTCCCGGAGAA | 21 |
| STAT1-E5R | GATCTTTCTTAAAGCCTGGTTC | 22 |
| STAT1-E5RS | TTCATCATTGCTTTGACATGGG | 22 |
| STAT1-E6F | ATAAGCAGGAAGCCTGTAGTAC | 22 |
| STAT1-E6R | ACTTATAGCTTGAGACTTCTGCA | 23 |
| STAT1-E7F | AGAACTGTCCAAGTCAGATCTC | 22 |
| STAT1-E7R | GGCCTGGGTTATCAAGGAAG | 20 |
| STAT1-E8F | AAGCCAAACTTTGACCTGTCAC | 22 |
| STAT1-E9R | GAAGCTGGACTATGTCAAACTC | 22 |
| STAT1-E10F | CTTTAATCCAGGCTGCTTCTGGAC | 24 |
| STAT1-E10R | CCTATTAAACCCTTGTAAATCATC | 24 |
| STAT1-E11F | ACTTTGTTCATCCAACTTGTCAG | 23 |
| STAT1-E11R | CTCTCAGATATTCTCAGTAAGAG | 23 |
| STAT1-E12F | CCTAAAACTGGAGGGGGAGTAG | 22 |
| STAT1-E12R | TCCACCCAGTATAGACCCTTC | 21 |
| STAT1-E13F | CATTCACTTACACTCTTATGCTC | 23 |
| STAT1-E14R | ACAAAGTCTACAAACCCCAGCA | 22 |
| STAT1-E15F | CCAATTTGTCCCATGTTCTGCA | 22 |
| STAT1-E15R | TCCTTTGCTGCTCTTCCCTGA | 21 |
| STAT1-E16F | GAGTCTTCAGACTTGCCACTGA | 22 |
| STAT1-E16R | ACCTCCAGAACAAACACTGAGA | 22 |
| STAT1-E17F | TAAACTGGACAGAAGAACAGAAC | 23 |
| STAT1-E17R | CCACAGGAGCTTTGTCACTTC | 21 |
| STAT1-E18F | CCTACTGTGAAAGCACCTGTG | 21 |
| STAT1-E18R | AAATAGCAGAGGGGAAAAGAGC | 22 |
| STAT1-E19F | ATCTTTGTTGGGTGTTTGGCTC | 22 |
| STAT1-E19R | CCAACCTCCTGCACTGAAGAA | 21 |
| STAT1-E20F | CTGTGCTGAATGGGACAGTTCCA | 23 |
| STAT1-E20R | ATGCGCACTCCTGTGAGATTC | 21 |
| STAT1-E21F | CTCACATTCCAGCCATTTTCTTG | 23 |
| STAT1-E21R | TTTGGGGTAAGTATAAGATCTGC | 23 |
| STAT1-E22F | GAGGTTCACTCAAATCCATCAAC | 23 |
| STAT1-E22R | CTTACTAGCTGTATCAGGCCAA | 22 |
| STAT1-E23F | CAGGCAGGGAGGTTATAGCTC | 21 |
| STAT1-E23R | AATGCTGATAGGCAGTAACACG | 22 |
| STAT1-E24F | TACAAGTGTATGCACTTACCCAA | 23 |
| STAT1-E24R | ATGGCTCATCTGAGCACTGCA | 21 |
| STAT1-E25F | AAGGCCCTCTTATTCCACGGTATTTA | 26 |
| STAT1-E25R | CAATGGAAAACTGCCAGTTACAC | 23 |
