# Supplementary material for: Application of Flow Cytometry in the Diagnostics Pipeline of Primary Immunodeficiencies Underlying Disseminated Talaromyces marneffei Infection in HIV-Negative Children
Source: Front Immunol. 2019 Sep 13;10:2189. doi: 10.3389/fimmu.2019.02189 (PMC6753679; doi:10.3389/fimmu.2019.02189)

Supplementary Fig. 1

Gating strategy for intracellular phosphorylated STAT1 (pSTAT1) in CD3+ T-cells and CD14+ T-cells by flow cytometry. Representative flow plots shown for patient F6.2 and a healthy control.

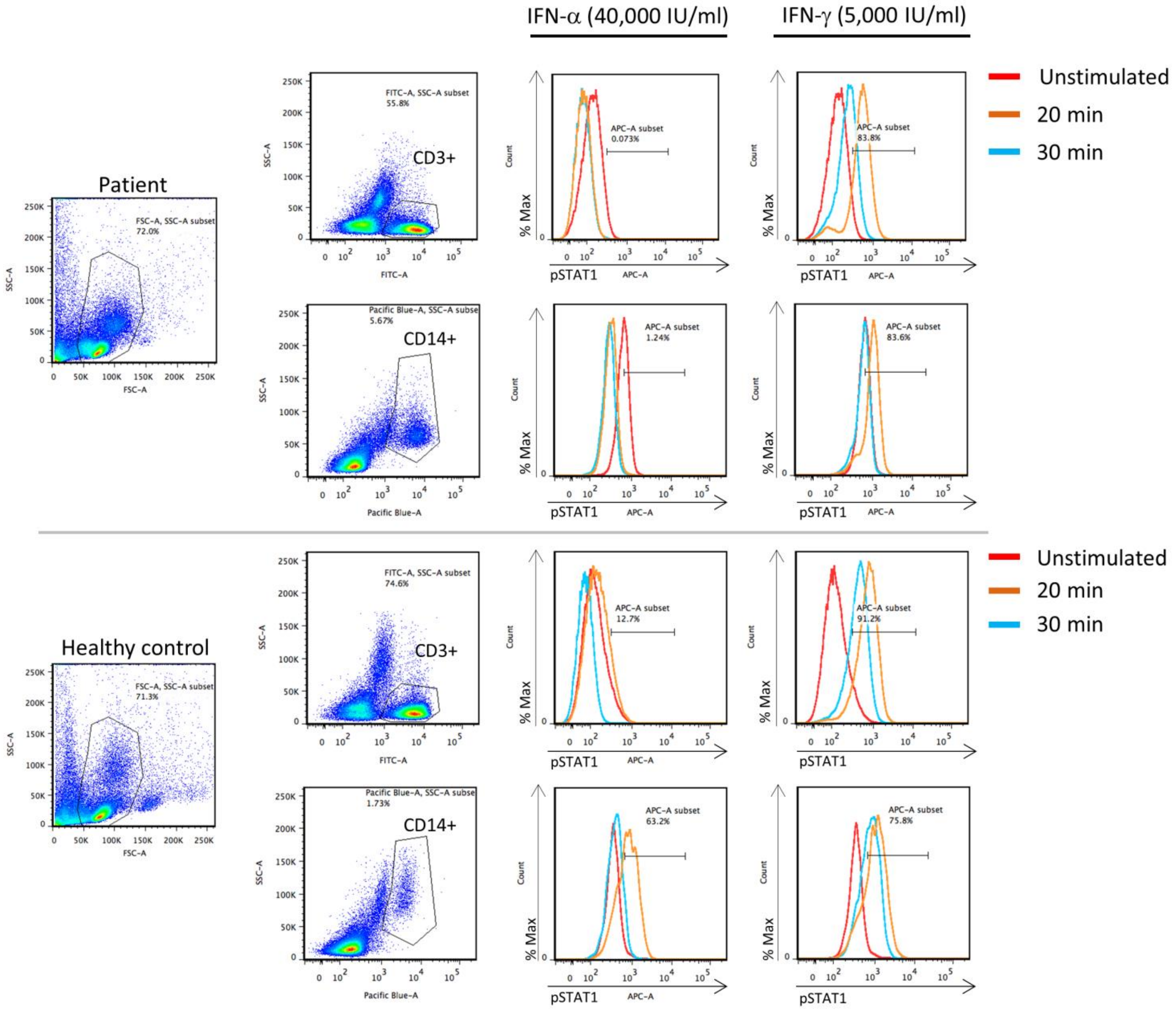

Supplement: Supplementary file 2 [file Image_1.pdf]
